# Female Faculty Representation in Anesthesiology: A Retrospective Cross-Sectional Analysis

**DOI:** 10.7759/cureus.75045

**Published:** 2024-12-03

**Authors:** Joseph Novoa, Skylar R Harmon, Saket Pandit, Kiranjit Kaur, Anastassia Shifchik, Saeeda Dhanani, Cristina Benites, Michelle Demory Beckler

**Affiliations:** 1 Transitional Year, HCA Florida Westside Hospital, Plantation, USA; 2 Allopathic Medicine, Nova Southeastern University Dr. Kiran C. Patel College of Allopathic Medicine, Davie, USA; 3 Transitional Year, HCA Florida Citrus Hospital, Inverness, USA; 4 Microbiology and Immunology, Nova Southeastern University Dr. Kiran C. Patel College of Allopathic Medicine, Fort Lauderdale, USA

**Keywords:** anesthesiology residency, female faculty, freida, women in anesthesiology, women in medicine

## Abstract

Introduction: The presence of female faculty members in anesthesia residency programs is pivotal for enhancing and supporting diversity and gender equity in medical education. This study probes the intricate interplay between the percentage of female faculty and whether program type and geographical location impact the composition of female faculty.

Methods: For this retrospective cross-sectional analysis, we collected data from the Fellowship and Residency Electronic Interactive Database Access (FREIDA) system to assess the percentages of female faculty in anesthesia residency programs. Stratification was based on program type: university-based, community-based/university-affiliated, and community-based. Regions were categorized using the Association of American Medical College (AAMC) geographical signaling divisions. Analysis of variance (ANOVA) was employed to gauge geographic variation in female faculty percentages across program types.

Results: Data on the percentage of female faculty were available for 89 programs. Among these, the average percentage of female faculty positions was 34%, ranging from 14% to 59%. Importantly, our analysis found no statistically significant differences in the distribution of female faculty positions based on program type or geographical location (p > 0.05).

Conclusions: This study reveals that the percentage of female faculty in anesthesia residency programs remains consistent regardless of program type or geographical location. These findings advance our understanding of gender dynamics within anesthesia residency faculties and underscore the need to explore additional factors influencing gender representation in medical education.

## Introduction

The involvement of women in the field of medicine in anesthesiology has been rising gradually over the last decade. Research examining female representation in the field has shown an upward trend since the early 2010s. This move towards gender diversity is important for promoting equitable representation within the medical field and contributing to diversity amongst physicians.

Although the number of female medical school graduates has been increasing, their representation within academic faculties has not kept pace [1]. This gap is noticeable in anesthesiology training programs where there are fewer female educators, with only 37% of teaching positions in anesthesiology held by women [2-4]. While the number of female program directors has risen from 15% in 2003 to 36% in 2018, achieving equal representation with male counterparts remains a challenge [5]. This imbalance raises questions about what factors affect women achieving teaching positions within anesthesiology departments, particularly in relation to program types, location, and institutional practices.

This review aims to understand why women are underrepresented in leadership roles within the field of anesthesiology and fill existing gaps in knowledge on this issue. By examining the relationship between the percentage of female faculty in anesthesia residency programs and factors such as program type and geographic variations, this cross-sectional analysis aims to provide insight that could contribute to a more representative and equitable environment for female faculty within anesthesiology residency programs.

## Materials and methods

Study design

This study used a retrospective cross-sectional design to assess the representation of female faculty in anesthesia residency programs across the United States. The analysis was based on data extracted from the Fellowship and Residency Electronic Interactive Database Access (FREIDA) system, which serves as an open resource for information on graduate medical education programs. This method allowed for an examination of the existing data to identify patterns and differences in female faculty representation based on program type and location.

Study population

This study focused on anesthesia residency programs in the United States. The programs were categorized into three types and divided based on their affiliation: university-based, community-based/university-affiliated, and community-based programs. This categorization was used to facilitate stratified analyses and comparisons among different institutional settings.

Geographic and program stratification

Geographic analysis was carried out using signaling divisions from the Association of American Medical Colleges (AAMC). These divisions served as a framework for examining disparities in female faculty representation taking into account variations in medical education resources and demographic characteristics across the country. This stratification allowed for a detailed analysis of regional variations in the percentage of female faculty members.

Outcome measure

The primary outcome was the percentage of female faculty within each anesthesia residency program. This percentage was analyzed for each program and compared across different program types and geographical regions.

Statistical analysis

Data were analyzed using a two-way analysis of variance (ANOVA) test to evaluate differences in the percentages of female faculty across the three program types and the AAMC-designated geographic regions. ANOVA was chosen due to its ability to assess mean differences between multiple groups while controlling for variability within each group. Post-hoc analyses were conducted as necessary to further explore significant differences identified by the ANOVA. All statistical analyses were performed using R (v4.3.1; R Development Core Team, Vienna, Austria), with a Bonferroni-adjusted significance level of 0.016 (0.05/3).

Ethical considerations

Given the cross-sectional nature of this study and the use of publicly available data from the FREIDA database, no patient data were involved, and ethical approval was not required. The study adhered to all guidelines regarding ethically handling data for research purposes.

## Results

Our analysis included 89 accredited allopathic anesthesia residency programs, classified into three categories: 64 (72%) university-based, 19 (21%) community-based/university-affiliated, and six (7%) community-based. A regional analysis was also conducted, and the regions investigated in this analysis are shown in Table 1. Data on female faculty percentages were available for 89 of these programs. The average percentage of female faculty across these programs was 34.1%, with a range spanning from 14.2% to 59.2%.

**Table 1:** Summary of regional distribution of programs

Region	Number (% of total)
East North Central	18 (20%)
East South Central	6 (7%)
Middle Atlantic	20 (23%)
Mountain	2 (2%)
New England	10 (11%)
Pacific	6 (7%)
South Atlantic	14 (16%)
West North Central	4 (5%)
West South Central	9 (10%)

**Figure 1:**
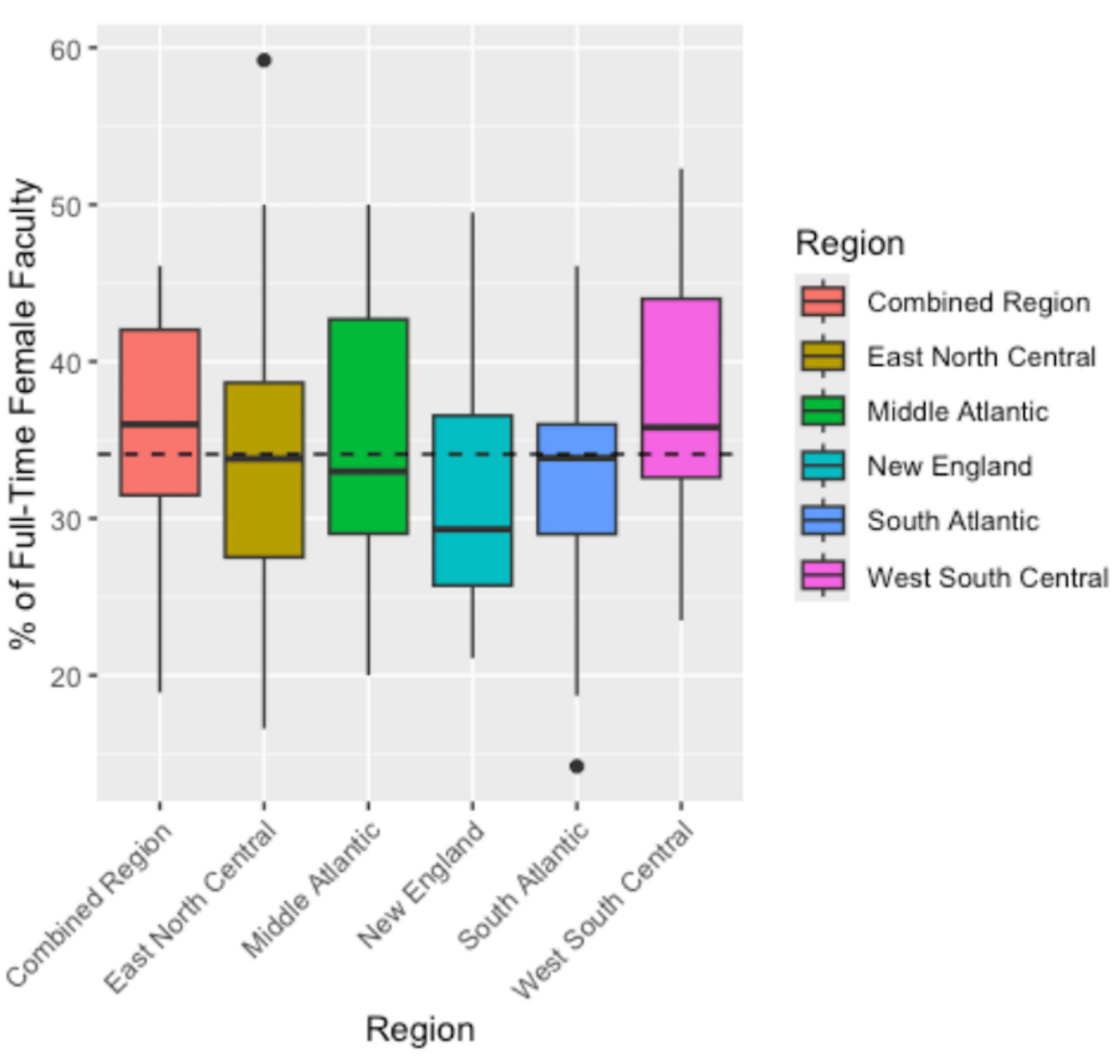
Boxplot illustrating the regional variation in female faculty percentage

**Figure 2:**
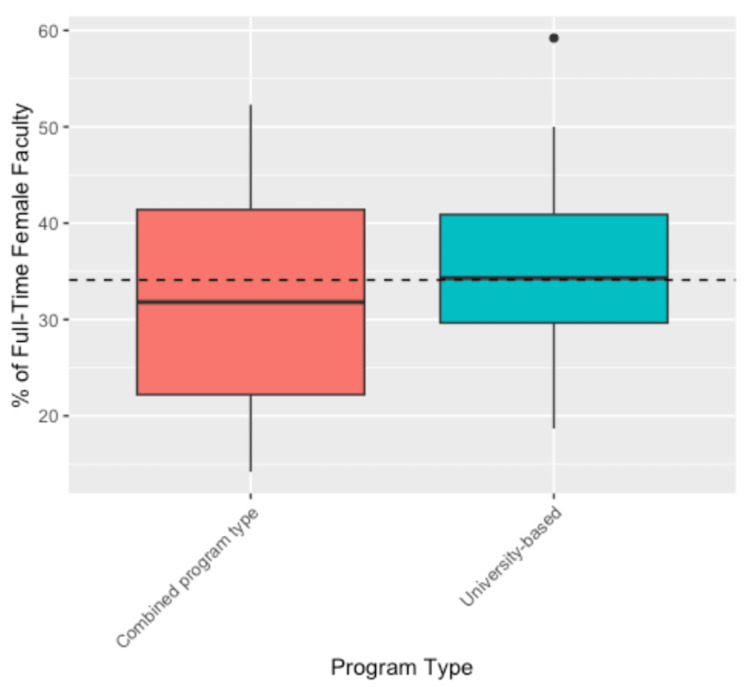
Boxplot illustrating the variation in the percent of female faculty by program type

A two-way ANOVA was conducted to examine the effects of region and program type on the percentage of full-time paid female faculty across residency programs. Due to the low number of programs in certain regions (East South Central, Mountain, Pacific, West North Central) and of certain program types (community-based, community-based university-affiliated), we combined these into a single “combined region” and “combined program type,” respectively. The results of this analysis are shown in Table 2. The main effect of region was not statistically significant (F(5, 77) = 0.755, p = 0.585). Similarly, the main effect of the program type was not statistically significant (F(1, 77) = 1.453, p = 0.232). Moreover, the interaction effect between region and program type was also not statistically significant (F(5, 77) = 0.492, p = 0.781), indicating that the effect of region on female faculty percentage did not depend on the program type. The 97.5% confidence interval estimates for the true mean percentage of female faculty by region and program type are shown in Tables 3 and 4, respectively. Figures 1-2 visualize the distribution of the percentage of female faculty in residency across different regions and program types, respectively.

**Table 2:** Two-way ANOVA to investigate the impact of both region and program type on female faculty percentage

Source	D.f.	Sum Sq	Mean Sq	F value	p
Region	5	324	64.84	0.755	0.585
Program type	1	125	124.72	1.453	0.232
Region:Program type	5	211	42.23	0.492	0.781
Residuals	77	6611	85.86	-	-

**Table 3:** 97.5% confidence interval estimates for the true mean female faculty percentage, by region

Region	Estimated Mean	Std Error	Lower	Higher
East North Central	33.9	2.24	28.8	39.0
Middle Atlantic	34.2	2.90	27.5	40.8
New England	31.1	2.99	24.2	37.9
South Atlantic	30.4	2.58	24.4	36.3
West South Central	38.0	3.28	30.5	45.5
Combined Region	34.2	2.93	27.5	40.9

**Table 4:** 97.5% confidence interval estimates for the true mean female faculty percentage, by program type

Program Type	Estimated Mean	Std Error	Lower	Higher
Combined type	32.3	1.95	27.8	36.7
University-based	35.0	1.26	32.1	37.9

## Discussion

This study investigates the relationship between the percentage of women faculty in anesthesia residency programs and factors such as program type and geographical location, a finding that women's representation on faculty remains consistent across different types of programs and locations. These findings advance our understanding of gender dynamics within anesthesia departments and underscore the need to explore additional factors influencing gender representation in medical education.

While the percentage of women in medical schools has grown from previous years with the total graduates by United States MD granting medical schools comprising 51.8% women in the 2022-2023 school year, women continue to be underrepresented in the field of anesthesiology. In 2013, women comprised up to 36% of anesthesiology trainees, and, in 2019, only 33% showed a downward trend [6]. This is clinically significant as diversity is crucial in United States healthcare where patients comprise a wide range of ethnic and racial backgrounds. Harbell et al. [7] recently illustrated that, in anesthesiology, women have a higher proportion of leadership roles in anesthesiology societies (32.6%) than the proportion of women in the anesthesia workforce (26%). This was not true for academic leadership roles where women remain underrepresented [8].

An important consideration is that medicine has a significant economic impact on family planning. The average age of entering medical students per the 2022 Matriculating Student Questionnaire from the AAMC is between 23 and 25 years old, setting expected graduation from 27 to 31 years old [9]. Female fertility has been shown to gradually decrease in the early 30s with a substantial drop starting at age 37, making child-bearing an important consideration during both residency and fellowship training periods [10]. Based on the United States Department of Agriculture 2015 Expenditures on Children by Families and an inflation rate of 25.6% from 2015 to 2023, the average cost of raising a child in the United States is about $15,512.52-$17,459.43 per year [11]. For resident physicians who have or plan to have children during training, this cost would take a significant portion of their income surplus. A 2018 survey of female members of the American Society of Anesthesiologists investigated challenges associated with being pregnant or having children during their training. Of the 752 total pregnancies, 23.3% responded yes or unsure that income loss related to parental leave with a child adversely affected their ability to financially support their family. The mean number of weeks of maternity leave among respondents was 8.1 with a mean of 4.4 of those weeks being paid and a mean of 2.8 weeks being used for additional vacation time among respondents [12].

One study showed that female faculty in anesthesiology experience a lower sense of workplace belonging, reduced self-efficacy for career advancement, and perceived less gender equity compared to their male counterparts [13]. Women are also less likely to believe their institutions are addressing diversity goals or are family-friendly. Additionally, there is less alignment between women’s values and those of their institutions. However, women and men show no significant differences in engagement, leadership aspirations, or perception of institutional efforts to support faculty advancement.

In the 2018 International Anesthesiology Issue: Women in Anesthesia: Challenges and Solutions, female academic leaders from anesthesiology shared their experiences and historical perspectives on the role of women in anesthesia. One author shares the perspective of the gender gap in academic promotion within anesthesiology and suggests the top things a woman must do when navigating this process [14]. Among these recommendations, knowing the impact of scholarly activities, seeking the right mentors, finding necessary support outside of academic medicine, advertising parenthood as a woman’s strength, not weakness, negotiation, and self-advocacy were among the top. In traditional academic models, success may depend on an uninterrupted commitment to the career, which causes long-lasting effects for women seeking faculty positions because the most intensive years of career building are more likely to coincide with childbearing years.

Furthermore, studies have shown gender differences in key aspects of academic promotion, including recommendation letters and evaluation ratings from students. One study investigating the influence of gender on student evaluations found that, when the instructor's gender was blinded, the identity perceived as male received significantly higher ratings than the identity perceived as female, regardless of the instructors' actual genders [15]. Another study found that recommendation letters written by both men and women exhibited “pervasive, systemic gender biases” [16]. Letters recommending women applicants were generally shorter and emphasized personality traits, while those written for men more often included details about their aptitudes and abilities in the workplace.

Study limitations 

This study is limited by the number of programs investigated. Over 40% of programs in our original dataset did not have any data regarding the percentage of full-time female faculty numbers. As a result, we have an over-representation of university-based programs in this analysis. More data are necessary in order to provide conclusive evidence that gender bias, as measured by the percentage of full-time female faculty members, does not exist between program types.

## Conclusions

This research explores the factors that affect the gender gap in faculty within residencies, particularly anesthesiology residencies, to enhance fairness in training and education practices. Our findings show that the presence of women in anesthesia residency faculties is consistent across program types and regions compared to the wider gender disparities observed in other medical fields. Future studies should focus on exploring factors affecting gender diversity in medical education to enhance inclusivity and promote equality, within anesthesiology residency programs.
